# S132. A NORMATIVE CHART FOR THE TRAJECTORY OF COGNITIVE FUNCTIONING IN INDIVIDUALS AT HIGH RISK FOR SCHIZOPHRENIA: LONGITUDINAL FINDINGS FROM THE INTERNATIONAL BRAIN AND BEHAVIOR CONSORTIUM ON 22Q11.2 DELETION SYNDROME

**DOI:** 10.1093/schbul/sby018.919

**Published:** 2018-04-01

**Authors:** Fiksinski Ania, Jacob Vorstman, Anne Bassett, Elemi Breetvelt

**Affiliations:** 1 UMC Utrecht; 2 UMC Utrecht, University of Toronto; 3 University of Toronto

## Abstract

**Background:**

In schizophrenia, a decline in cognitive functioning often precedes the onset of the first psychotic episode by many years. We have previously shown this to be the case for individuals with 22q11.2 deletion syndrome (22q11DS), a genetic variant associated with a 20–25% risk of developing schizophrenia. We also observed that, regardless of the subsequent development of psychosis, individuals with 22q11DS show, on average a modest decline in IQ between 8 and 24 years and that in those who develop a psychotic disorder this decline follows a steeper trajectory. The tendency for a modest cognitive decline may represent a cognitive phenotype that is specific to 22q11DS, and possibly, an endophenotype for schizophrenia in this population. In order to assess this, we constructed a normative chart for cognitive development over time in individuals with 22q11DS.

**Methods:**

We made use of the International Brain and Behavior Consortium on 22q11DS (IBBC) database that includes cross-sectional and longitudinal cognitive data from 1871 individuals with 22q11DS (mean age 15.7, SD 7.4, years; n = 330 (17.6%) with schizophrenia). All IQ measurements were obtained by qualified personnel using Wechsler tests, including WPPSI, WISC and WAIS, depending on the participants’ ages. We used all available IQ data points obtained in the age range 6 - 40 years to construct normative charts for Full Scale IQ (FSIQ), Verbal IQ (VIQ) and Performance IQ (PIQ), after a comprehensive quality control procedure to ensure reliable and comparable IQ data across all international sites. We used a polynomial regression model, similar to what is used in standardized international growth charts for height and weight, to create the normative chart.

**Results:**

The 4th order polynomial regression provided a good fit for the IQ data from our sample, allowing for the observed larger variability in very young age groups. On average, between the ages of 6 and 40 years, the 22q11DS population showed a gradual, modest decline in FSIQ, VIQ and PIQ. Consistent with our previous results, individuals who went on to develop schizophrenia showed a steeper decline, representing a deviation from their expected trajectory, than those who had no documented psychotic illness.

**Discussion:**

This study is, to our knowledge, the first to demonstrate that a normative chart for cognitive development through to adulthood can be reliably constructed for a genetically selected high-risk population. This normative chart can be readily applied both in clinical care and in research and may serve as an example for constructing similar normative charts in other high-risk groups and/or genetic disorders.

